# First report outside Eastern Europe of West Nile virus lineage 2 related to the Volgograd 2007 strain, northeastern Italy, 2014

**DOI:** 10.1186/s13071-015-1031-y

**Published:** 2015-08-13

**Authors:** Silvia Ravagnan, Fabrizio Montarsi, Stefania Cazzin, Elena Porcellato, Francesca Russo, Manlio Palei, Isabella Monne, Giovanni Savini, Stefano Marangon, Luisa Barzon, Gioia Capelli

**Affiliations:** Istituto Zooprofilattico Sperimentale delle Venezie, Legnaro, Padova Italy; Promotion and Development of Hygiene and Public Health, Veneto Region Venezia, Italy; Veterinary Public Health Service, Friuli Venezia Giulia Region Udine, Italy; Istituto Zooprofilattico Sperimentale dell’Abruzzo e del Molise, Teramo, Italy; Department of Molecular Medicine, University of Padova, Padova, Italy

**Keywords:** West Nile virus lineage 2, Volgograd strain, *Culex pipiens* s.l, Northeastern Italy

## Abstract

**Background:**

West Nile virus (WNV) is a Flavivirus transmitted to vertebrate hosts by mosquitoes, maintained in nature through an enzootic bird-mosquito cycle. In Europe the virus became of major public health and veterinary concern in the 1990s. In Italy, WNV re-emerged in 2008, ten years after the previous outbreak and is currently endemic in many areas of the country. In particular, the northeastern part of Italy experience continuous viral circulation, with human outbreaks caused by different genovariants of WNV lineage 1, Western-European and Mediterranean subcluster, and WNV lineage 2, Hungarian clade. Alongside the WNV National Surveillance Program that has been in place since 2002, regional surveillance plans were implemented after 2008 targeting mosquitoes, animals and humans.

**Findings:**

In July and September 2014, West Nile virus lineage 2 was detected in pools of *Culex pipiens* s.l. mosquitoes from northeastern Italy. Whole genome sequencing and phylogenetic analysis of two representative samples identified the presence of WNV lineage 2 related to the Volgograd 2007 strain (99.3 % nucleotide sequence identity), in addition to WNV lineage 2 Hungarian clade.

**Conclusions:**

This is the first evidence of the circulation of a WNV lineage 2 strain closely related to the Volgograd 2007 outside Eastern Europe, where it has caused large human outbreaks. This strain may pose a new threat to animal and human health in Italy.

## Background

West Nile virus (WNV) is a Flavivirus transmitted to vertebrate hosts by mosquito species, mainly of the genus *Culex*, and is maintained in nature through an enzootic bird-mosquito cycle [1]. Although they may develop fatal disease, horses and humans are considered dead-end hosts. In Europe the virus has been circulating since the 1960s, but only became of major public health and veterinary concern in the 1990s [2]. In Italy, WNV appeared for the first time in 1998 in horses of the Tuscany region [3]. In 2008, ten years after the previous outbreak, it re-emerged in northern Italy and is currently endemic in many areas of the country [4]. In particular, Veneto and Friuli Venezia Giulia (FVG) regions, in the northeastern part of Italy, experience continuous viral circulation, with the occurrence of human outbreaks caused by different genovariants of WNV lineage 1 (WNV lin.1), Western-European and Mediterranean subcluster, and WNV lineage 2 (WNV lin.2), Hungarian clade [5–7]. Alongside the WNV National Surveillance Program that has been in place since 2002, regional surveillance plans were implemented after 2008 targeting mosquitoes, animals and humans [8–10]. Here we report the first evidence of circulation in Italy of WNV lin.2 related to the Russian strain Volgograd 2007 in mosquitoes collected during the entomological surveillance conducted in 2014. The Volgograd strain has been associated with large outbreaks in horses and in humans in the Volgograd and Astrakhan regions since 2004 [11] and in Romania since 2010 [12, 13], but, so far, has never been reported outside Eastern Europe.

## Findings

From May to October 2014, 39 entomological CDC-CO_2_ traps (Centers for Disease Control and Prevention trap like, baited with carbon dioxide) (IMT, Italian Mosquito Trap, Cantù, Italy) were operated in Veneto and FVG regions for one night every 15 days. Collected mosquitoes were identified, pooled (50 specimens maximum) according to date and site and screened for Flavivirus by using a one-step SYBR Green-based Real-Time RT-PCR targeting 250 bp of the conserved region of the non-structural NS5 gene [7]. To confirm the presence of WNV, all Flavivirus-positive samples were tested by an RT-PCR targeting 705 bp of the NS5 gene [7].

Complete genomes of two WNV strains were sequenced and submitted to the GenBank database (Accession numbers KT207791-KT207792). Oligonucleotide primers used to generate the complete sequences are available upon request. Phylogenetic analyses were carried out using the neighbour-joining method with 1,000 bootstrap replicates implemented in the MEGA 5 program [14].

Overall, 91,779 mosquitoes were collected in 2014, mostly represented by *Cx. pipiens* s.l. (85 %). Mosquitoes collected according to species, pools tested and pools positive for WNV are reported in Table 1.

**Table 1:** Mosquito species collected in 2014 in Veneto and Friuli Venezia Giulia regions, pool tested and pool positive for WNV

Species	Tot. collected	Percent	Pool tested	Pool pos. WNV (%)
*Culex pipiens* s.l.	78,131	85.129	1,514	9 (0.59)
*Ochlerotatus caspius*	10,290	11.212	301	0
*Aedes albopictus*	1,453	1.583	0	-
*Aedes vexans*	932	1.015	0	-
*Anopheles maculipennis* s.l.	416	0.453	0	-
*Anopheles plumbeus*	320	0.349	0	-
*Aedes/Ochlerotatus* spp.	119	0.130	2	0
*Culiseta annulata*	41	0.045	0	-
*Ochlerotatus cantans*	21	0.023	0	-
*Ochlerotatus geniculatus*	13	0.014	0	-
*Culiseta longiareolata*	12	0.013	0	-
*Coquillettidia richiardii*	11	0.012	0	-
*Culex modestus*	7	0.008	4	0
*Ochlerotatus detritus*	7	0.008	0	-
*Anopheles claviger/petragnani*	3	0.003	0	-
*Ochlerotatus sticticus*	2	0.002	0	-
*Ochlerotatus echinus*	1	0.001	0	-
Total	91,779	100	1,821	9 (0.49)

**Fig. 1:**
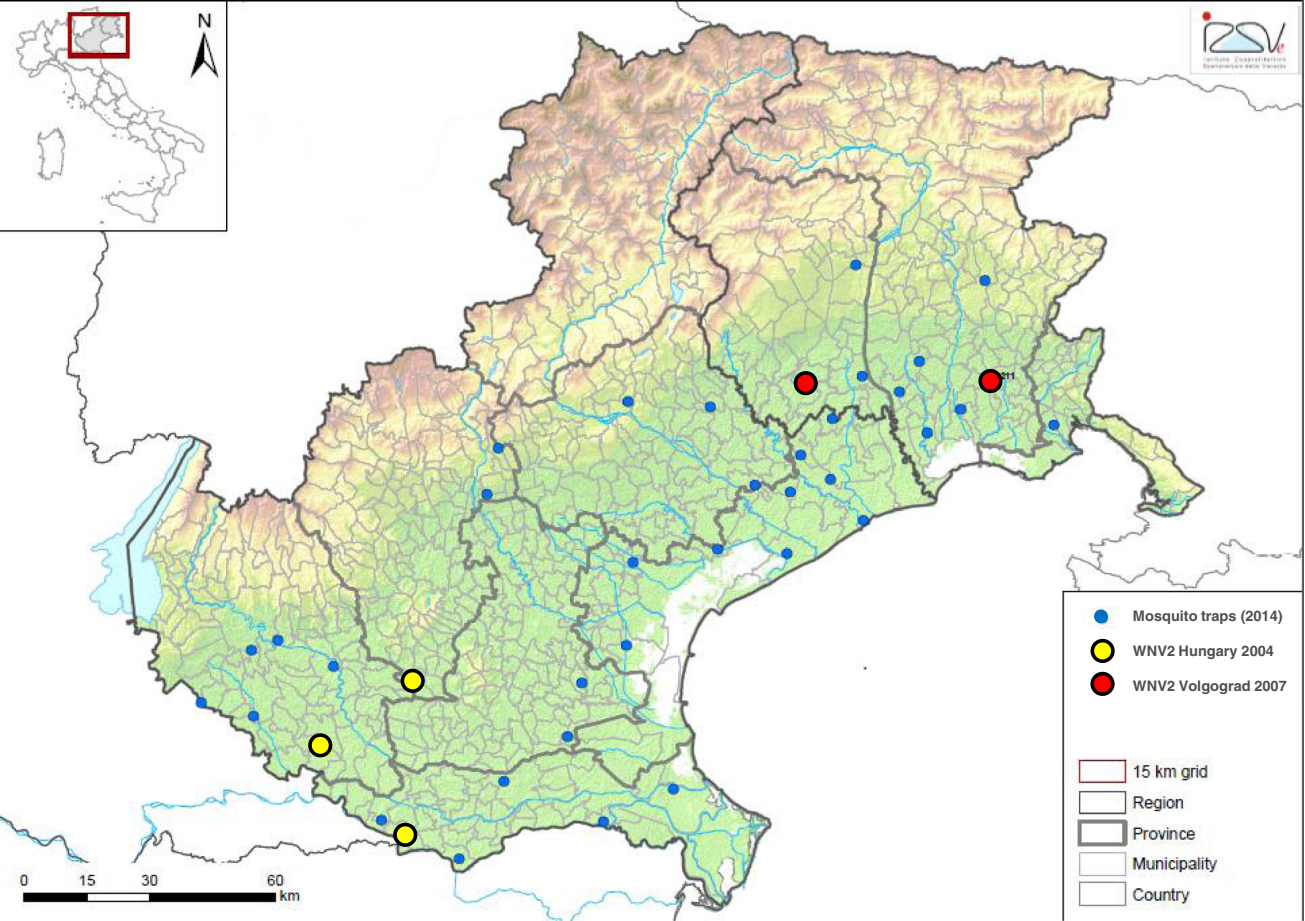
Map showing the location of the entomological traps in 2014 in northeastern Italy and the sites of *Culex pipiens* s.l. mosquitoes positive for WNV lin.2 Volgograd-like (red) and Hungary-like strains (yellow)

**Fig. 2:**
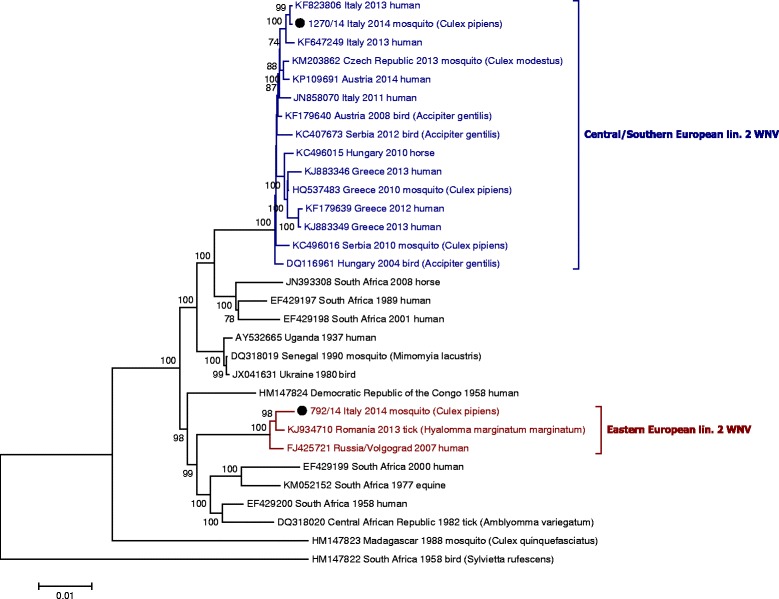
*Phylogenetic tree of WNV lineage 2 complete sequences. Legend:* Sequence dataset was analysed using MEGA5, the neighbour-joining (NJ) method, and bootstrap analysis (1,000 replicates) based on the ClustalW algorithm. Significant bootstrapping values (>70 %) are shown on the nodes. (●) Italian WNV lineage 2 genomes detected in pools of *Culex pipiens* s.l. in northeastern Italy

WNV lin.2 was detected in *Culex pipiens* s.l. from five sites, three along the Po river (Veneto region) and two further north, close to the Grado-Marano lagoon (FVG region) (Fig. 1). The preliminary phylogenetic analysis of the NS5 partial sequences suggested that the viruses detected in the two regions corresponded to two genetically diverse groups. The whole genome sequences and phylogenetic analyses of two representative samples showed that the WNV lin.2 from Veneto region clustered with those circulating in Italy in the previous years and belonged to the Hungary/04 cluster, while the WNV lin.2 from FVG region was closely related to the Russian strain Volgograd 2007 (99.3 % nucleotide sequence identity) (Fig. 2).

The mosquitoes infected with WNV lin.2 strain similar to Volgograd 2007 were collected on July 15th and September 16th in two sites 50 km apart (Fig. 1). In the surrounding area no human or veterinary cases were notified, however, serological evidence of viral circulation was confirmed by the National Centre for Exotic Diseases (CESME) in two horse farms [15].

This report is the first evidence of the circulation of Volgograd 2007 WNV lin.2 like strain outside Eastern Europe. Phylogeography analysis suggested that WNV lin.2 might have been introduced independently in Hungary (WNV lin.2 strains of the Hungarian clade) and Russia (WNV lin.2 Volgograd 2007 strain) at the end of the 20th century by migratory birds through different migratory bird routes from Africa [16]. After the first report in birds in Hungary in 2004, the WNV lin.2 Hungarian clade explosively emerged in 2008/2009 in Hungary and Austria, before expanding into Southern Europe, reaching Greece in 2010 through the Balkans [17]. Recently it has also been found in mosquitoes in the Czech Republic, 2013, suggesting a possible expansion toward northern Europe [18].

In Italy, WNV lin.2 strains belonging to the Hungarian clade were first detected in 2011 in the northeastern regions, in particular in mosquitoes collected in FVG and in birds from Veneto [5]. Two human cases were also notified in 2011 in Central Italy and Sardinia [19, 20]. In 2013, WNV lin.2 strains were responsible for transmission of WNV in northern Italy, with 28 human cases reported [6].

The WNV lin.2 strain related to Volgograd 2007 detected in northeastern Italy represents a new independent introduction of WNV in a small geographic area, where other introductions had occurred in the previous years. For instance, the WNV lin.1 strains of the Western European-Mediterranean subcluster in 2008 and 2011, and WNV lin.2 strains of the Hungarian clade in 2011 [5, 7, 21].

The WNV lin.2 (Volgograd strain) was identified in Russia in 2004 in patients with neuroinvasive disease [11]. This strain was associated with large human outbreaks reported in the Volgograd area in 2007 and 2010 [11] and in Romania since 2010 [12, 13]. A closely related WNV lin.2 strain was identified in a tick collected from a bird in Romania in 2013 [22].

The area borders the Adriatic sea and is an important corridor for migratory birds, offering resting places in three main wetlands, part of the Po river Delta, the Venice lagoon and the Grado-Marano lagoon, which corresponds to the areas of the main human outbreaks in the past years. It is currently unknown if this strain was introduced with birds migrating from Africa or through a shorter migration from Eastern Europe.

This finding further complicates the already complex scenario of WNV circulation in northeastern Italy [7, 21]. The heterogeneity of WNV strains co-circulating in northeastern Italy and in other parts of Europe demonstrates the ability of this virus to spread and adapt to new habitats and hosts. This may also create difficulties in the prompt detection of currently circulating or newly emerging genotypes. The continual optimization of the sensitivity and specificity of the molecular and serological tools currently used toward lineages and strains of WNV is therefore recommended.

## Conclusions

A novel WNV lin.2 Volgograd 2007-like strain has been detected in northern Italy and may pose a new threat to animal and human health, similar to the occurrence in Eastern Europe in the last decade.

This finding provides further evidence that northeastern Italy is a crucial area for WNV introduction, circulation and spread, and exhorts to maintain and even reinforce the WNV integrated surveillance.

## References

[1] Hayes EB, Komar N, Nasci RS, Montgomery SP, O’Leary DR, Campbell GL (2005). Epidemiology and transmission dynamics of West Nile virus disease. Emerg Infect Dis.

[2] Zeller HG, Schuffenecker I (2004). West Nile virus: an overview of its spread in Europe and the Mediterranean basin in contrast to its spread in the Americas. Eur J Clin Microbiol Infect Dis.

[3] Autorino GL, Battisti A, Deubel V, Ferrari G, Forletta R, Giovanni A (2002). West Nile virus epidemic in horses, Tuscany region Italy. Emerg Infect Dis.

[4] Monaco F, Lelli R, Teodori L, Pinoni C, Di Gennaro A, Polci A (2010). Re-emergence of West Nile virus in Italy. Zoonoses Public Health.

[5] Savini G, Capelli G, Monaco F, Polci A, Russo F, Di Gennaro A (2012). Evidence of West Nile virus lineage 2 circulation in Northern Italy. Vet Microbiol.

[6] Barzon L, Pacenti M, Franchin E, Lavezzo E, Masi G, Squarzon L, et al. Whole genome sequencing and phylogenetic analysis of West Nile virus lineage 1 and lineage 2 from human cases of infection, Italy, August 2013. Euro Surveill. 2013;18(38). 10.2807/1560-7917.es2013.18.38.20591 24084339

[7] Capelli G, Ravagnan S, Montarsi F, Fabrizio S, Cazzin S, Bonfanti L (2013). Further evidence of lineage 2 West Nile Virus in *Culex pipiens* of North-Eastern Italy. Vet Ital.

[8] Gobbi F, Barzon L, Capelli G, Angheben A, Pacenti M, Napoletano G (2012). Surveillance for West Nile, dengue, and chikungunya virus infections, Veneto Region, Italy, 2010. Emerg Infect Dis.

[9] Mulatti P, Bonfanti L, Capelli G, Capello K, Lorenzetto M, Terregino C (2013). West Nile Virus in North-Eastern Italy, 2011: Entomological and Equine IgM-Based Surveillance to Detect Active Virus Circulation. Zoonoses Public Health.

[10] Barzon L, Pacenti M, Franchin E, Pagni S, Lavezzo E, Squarzon L (2013). Large human outbreak of West Nile virus infection in north-eastern Italy in 2012. Viruses.

[11] Platonov AE, Karan LS, Shopenskaia TA, Fedorova MV, Koliasnikova NM, Rusakova NM (2011). Genotyping of West Nile fever virus strains circulating in Southern Russia as an epidemiological investigation method: Principles and results (in Russian). Zh Mikrobiol Epidemiol Immunobiol.

[12] Sîrbu A, Ceianu CS, Panculescu-Gatej RI, Vázquez A, Tenorio A, Rebreanu R (2011). Outbreak of West Nile virus infection in humans, Romania, July to October 2010. Euro Surveill.

[13] Dinu S, Cotar AI, Pănculescu-Gătej IR, Fălcuţă E, Prioteasa FL, Sîrbu A (2015). West Nile virus circulation in south-eastern Romania, 2011 to 2013. Euro Surveill.

[14] Tamura K, Peterson D, Peterson N, Stecher G, Nei M, Kumar S (2011). MEGA5: molecular evolutionary genetics analysis using maximum likelihood, evolutionary distance, and maximum parsimony methods. Mol Biol Evol.

[15] West Nile disease Epidemiological Bulletin, 2014. Available online at: http://sorveglianza.izs.it/emergenze/west_nile/pdf/Bollettino_WND_04_05_2015_Riassuntivo_2014EN

[16] Ciccozzi M, Peletto S, Cella E, Giovanetti M, Lai A, Gabanelli E (2013). Epidemiological history and phylogeography of West Nile virus lineage 2. Infect Genet Evol.

[17] Bakonyi T, Ferenczi E, Erdélyi K, Kutasi O, Csörgö T, Seidel B (2013). Explosive spread of a neuroinvasive lineage 2 West Nile virus in Central Europe, 2008/2009. Vet Microbiol.

[18] Rudolf I, Bakonyi T, Sebesta O, Mendel J, Peško J, Betášová L (2014). West Nile virus lineage 2 isolated from *Culex modestus* mosquitoes in the Czech Republic, 2013: expansion of the European WNV endemic area to the North?. Euro Surveill.

[19] Bagnarelli P, Marinelli K, Trotta D, Monachetti A, Tavio M, Del Gobbo R (2011). Human case of autochthonous West Nile virus lineage 2 infection in Italy, September 2011. Euro Surveill.

[20] Magurano F, Remoli ME, Baggieri M, Fortuna C, Marchi A, Fiorentini C (2012). Circulation of West Nile virus lineage 1 and 2 during an outbreak in Italy. Clin Microbiol Infect.

[21] Barzon L, Pacenti M, Franchin E, Squarzon L, Lavezzo E, Cattai M (2013). The complex epidemiological scenario of West Nile virus in Italy. Int J Environ Res Public Health.

[22] Kolodziejek J, Marinov M, Kiss BJ, Alexe V, Nowotny N (2014). The Complete Sequence of a West Nile Virus Lineage 2 Strain Detected in a *Hyalomma marginatum marginatum* Tick Collected from a Song Thrush (*Turdus philomelos*) in Eastern Romania in 2013 Revealed Closest Genetic Relationship to Strain Volgograd 2007. PLoS One.

